# Supplementary material for: Empirical methods for controlling false positives and estimating confidence in ChIP-Seq peaks
Source: BMC Bioinformatics. 2008 Dec 5;9:523. doi: 10.1186/1471-2105-9-523 (PMC2628906; doi:10.1186/1471-2105-9-523)
Supplement: Additional File 1 — A variety of html documents from the USeq web site detailing the available applications, their best usage, output file type descriptions, command line menus, etc. [file 1471-2105-9-523-S1.zip › USeqUserGuides/JavaDoc.zip!/JavaDoc/trans/cel/package-frame.html]

trans.cel


trans.cel

Classes 
  
AverageACels
  
AveragePCels
  
CelFileConverter
  
CelLine
  
CelMapper
  
CelPHistogram
  
CelProcessor
  
ChromosomeIndex
  
ComparatorMultiSetQuantile
  
ComparatorQuantile
  
ControlStats
  
ConvertTVSToCelps
  
MakeChromosomeSets
  
MultiSetQuantile
  
Quantile
  
QuantileNormalization
  
RotateCelFile
  
SplineScalar
  
StatIntensityFeatureArray
